# Corilagin Restrains NLRP3 Inflammasome Activation and Pyroptosis through the ROS/TXNIP/NLRP3 Pathway to Prevent Inflammation

**DOI:** 10.1155/2022/1652244

**Published:** 2022-10-17

**Authors:** Tianyu Luo, Xiaoyi Zhou, Minyan Qin, Yuqing Lin, Jiefen Lin, Guangpei Chen, Aijun Liu, Dongyun Ouyang, Dongfeng Chen, Hao Pan

**Affiliations:** ^1^Department of Human Anatomy, School of Basic Medical Sciences, Guangzhou University of Chinese Medicine, Guangzhou, Guangdong, China; ^2^Research Center for Integrative Medicine, School of Basic Medical Sciences, Guangzhou University of Chinese Medicine, Guangzhou, Guangdong, China; ^3^Department of Immunobiology, College of Life Science and Technology, Jinan University, Guangzhou, Guangdong, China; ^4^Dongguan Institute of Guangzhou University of Chinese Medicine, Dongguan, Guangdong, China

## Abstract

Corilagin, a gallotannin, shows excellent antioxidant and anti-inflammatory effects. The NLRP3 inflammasome dysfunction has been implicated in a variety of inflammation diseases. However, it remains unclear how corilagin regulates the NLRP3 inflammasome to relieve gouty arthritis. In this study, bone marrow-derived macrophages (BMDMs) were pretreated with lipopolysaccharide (LPS) and then incubated with NLRP3 inflammasome agonists, such as adenine nucleoside triphosphate (ATP), nigericin, and monosodium urate (MSU) crystals. The MSU crystals were intra-articular injected to induce acute gouty arthritis. Here we showed that corilagin reduced lactate dehydrogenase (LDH) secretion and the proportion of propidium iodide- (PI-)stained cells. Corilagin suppressed the expression of N-terminal of the pyroptosis executive protein gasdermin D (GSDMD-NT). Corilagin restricted caspase-1 p20 and interleukin (IL)-1*β* release. Meanwhile, corilagin attenuated ASC oligomerization and speck formation. Our findings confirmed that corilagin diminished NLRP3 inflammasome activation and macrophage pyroptosis. We further discovered that corilagin limited the mitochondrial reactive oxygen species (ROS) production and prevented the interaction between TXNIP and NLRP3, but ROS activator imiquimod could antagonize the inhibitory function of corilagin on NLRP3 inflammasome and macrophage pyroptosis. Additionally, corilagin ameliorated MSU crystals induced joint swelling, inhibited IL-1*β* production, and abated macrophage and neutrophil migration into the joint capsule. Collectively, these results demonstrated that corilagin suppressed the ROS/TXNIP/NLRP3 pathway to repress inflammasome activation and pyroptosis and suggest its potential antioxidative role in alleviating NLRP3-dependent gouty arthritis.

## 1. Introduction

Corilagin (C_27_H_22_O_18_), a natural polyphenol tannic acid compound, is the main active component of *Phyllanthus urinaria*, *Phyllanthus emblica*, *Geranium*, and other medicinal plants [1]. Recent pharmacological researches have demonstrated that corilagin displays various biological activities, such as antioxidation [2] and anti-inflammation [3] activities. For example, corilagin can inhibit calcium oxalate crystal-evoked oxidative stress, inflammation, apoptosis, and relieve kidney stones in rats [4]. Corilagin enhances the survival rate of septic mice by inhibiting the expression of proinflammatory cytokines such as IL-1*β*, IL-6, and TNF-*α* [5]. Nevertheless, the mechanism of corilagin on anti-inflammation has not been fully elucidated.

The NLR family pyrin domain-containing protein 3 (NLRP3) inflammasome is deeply involved in the regulation of inflammation-related diseases [6]. The NLRP3 inflammasome is mainly a multimeric cytosolic protein complex composed of NLRP3, the adaptor protein apoptosis-associated speck-like protein containing a CARD (ASC) and the effector protease caspase-1. The classical activation of NLRP3 inflammasome comprises two steps. First, cells are primed via pattern recognition receptors, e.g., Toll-like receptor (TLR) 4 or tumor necrosis factor receptor (TNFR)), which activate the NF-*κ*B signaling to initiate *NLRP3* and *IL-1β* genes transcription [7]. Second, the NLRP3 inflammasome, composed of the NLRP3, ASC, and caspase-1, is activated by pathogen-associated molecular patterns (PAMPs) or damage-associated molecular patterns (DAMPs), e.g., ATP, nigericin, and MSU crystals. Subsequently, precursor (pro)-caspase-1 is cleaved into its active form (p10 and p20 subunits), which splits pro-IL-1*β* (31 kDa) to produce mature IL-1*β* (17 kDa) [7]. Gasdermin D (GSDMD) was also cleaved to obtain an N-terminal fragment (GSDMD-NT), which is an effector protein to induce pyroptosis and release cytokines [8]. Inflammation can eliminate foreign bodies and promote tissue repair, while sustained uncontrolled inflammation can engender tissue damage.

The NLRP3 can be activated by diverse molecules or cellular events, including mitochondrial dysfunction and mitochondrial DNA, ROS, ion flux, and lysosomal damage [9]. Upon the second signal stimulation, mitochondrial morphology changes and generates ROS. Excessive ROS causes thioredoxin (TRX) to dissociate from thioredoxin-interacting protein (TXNIP), and activated TXNIP combines with NLRP3 to promote inflammasome activation [10, 11]. The topical immunomodulator imiquimod induces NLRP3 activation by triggering ROS and mitochondrial complex I dysfunction [12]. However, it is unclear whether corilagin manages ROS generation to interfere with NLRP3 inflammasome activation.

Purine metabolism disorder causes urate accumulation to form MSU crystals, which can drive NLRP3 inflammasome oligomerization and IL-1*β* release and triggers acute gouty arthritis flares [13]. The NLRP3 senses MSU crystals mainly through the following two pathways. MSU crystals increase intracellular ROS production, which promotes TXNIP dissociation from TRX, thereby promoting NLRP3 inflammasome assembly. In addition, MSU crystals facilitate IL-1*β* secretion, which stimulates nonhematopoietic cells to generate IL-6 and chemokines to recruit neutrophils, resulting in inflammation and joint tissue damage [7, 14, 15]. Small molecular compounds derived from plants show promising potential in antigouty arthritis. Our previous research revealed that compounds derived from traditional Chinese medicine display potential therapeutic effects on NLRP3-dependent gouty arthritis [16, 17]. However, whether corilagin targets NLRP3 to treat gouty arthritis is unknown.

Here, we show that corilagin suppressed ASC polymerization, caspase-1 activation, mature IL-1*β* release, and macrophage pyroptosis. Importantly, corilagin blocked ROS-induced interaction between TXNIP and NLRP3. ROS activator imiquimod abrogated the function of corilagin in inhibiting NLRP3 inflammasome activation and macrophage pyroptosis. In addition, corilagin prevented joint swelling, reduced IL-1*β* and caspase-1 (p20) expression, and inhibited neutrophils and macrophages aggregation in the MSU crystals-caused arthritis mice. Our results illustrate that corilagin restricts ROS/TXNIP/NLRP3 pathway to diminish macrophage pyroptosis and proinflammatory cytokines release, thereby alleviating NLRP3-dependent gouty arthritis.

## 2. Materials and Methods

### 2.1. Mice

8-10 weeks-old C57BL/6 J male mice were came from the Laboratory Animal Center of Guangzhou University of Chinese Medicine. NLRP3-deficient (NLRP3^−/−^) mice, C57BL/6 J background, were kindly provided by Dr. Dongyun Ouyang of Jinan University, Guangzhou, China. All mice were maintained at a temperature- and humidity-controlled facility and fed in pathogen-free conditions. All animal studies were conducted by following the strict guidelines defined by the Institutional Animal Care and Use Committees of Guangzhou University of Chinese Medicine.

### 2.2. Reagents Resource

Corilagin (B20672) was acquired from Yuanye Bio-Technology (Shanghai, China). 20 mg corilagin was dissolved in 315 *μ*l DMSO to obtain a concentration of 100 mM, and then aliquoted 50 *μ*l into 6 centrifuge tubes and stored at -20°C. Adenosine triphosphate (ATP, A6419), fetal bovine serum (FBS, S8318), Hoechst 33342 (B2261), lipopolysaccharide (LPS, L4391), and propidium iodide (PI, P4170) were acquired from Sigma–Aldrich (St. Louis, MO, USA). Colchicine (T0320), H2DCFDA (T15458), and Imiquimod (T0134) were bought from TargetMol (Boston, MA, USA). Nigericin (tlrl-nig) and MSU crystals (tlrl-msu) were bought from InvivoGen (San Diego, CA, USA). MitoSOX™ Red (M36008), MitoTracker® Deep Red FM (M22426), and streptomycin/penicillin (15140122) were obtained from Thermo Fisher Scientific (CA, USA). The cell lysis buffer for western blot and IP (P0013), Enhanced Mitochondrial Membrane Potential Assay Kit with JC-1 (C2003S), Hematoxylin and Eosin (H&E) Staining Kit (C0105), and Lactate Dehydrogenase (LDH) Cytotoxicity Assay Kit (C0017) were purchased from Beyotime Biotechnology (Haimen, China). The BCA Protein Assay Kit (FD2001) and one-step gel preparation kit (FD341) were purchased from Fude Biological Technology (Hangzhou, China). Mouse TNF-*α* (EMC102a) and IL-1*β* (EMC001b) ELISA kits were purchased from Neobioscience Technology Co., Ltd. (Shenzhen, China). Histostain TM-Plus Kits (SP-0022) were from Bioss (Beijing, China). DAB Chromogenic Kit (G1212) was acquired from Servicebio Technology (Wuhan, China). Mayer's hematoxylin solution (G1080) was from Solarbio Science and Technology (Beijing, China). An ECL kit (picogram) (KF001) was obtained from Affinity Biosciences (Cincinnati, USA). Protein G agarose beads (#37478) and Protein A agarose beads (#9863) were obtained from Cell Signaling Technology (Danvers, MA). The antibody sources are shown in Table 1.

### 2.3. Cell Culture and Stimulation

L929 cells were incubated in high glucose DMEM supplemented with 10% FBS and 100 U/ml penicillin–streptomycin. The L929 cells culture medium was collected after the liquid was slightly yellow. The abundant M-CSF in the supernatant of L929 cells could induce mouse bone marrow-derived macrophages (BMDMs). In our previous studies, we established a mature method to generate BMDMs [16]. Briefly, bone marrow cells were collected and gently blown to disperse into a suspension. Then red blood cells were lysed, and the remaining bone marrow cells were incubated in high glucose DMEM containing 20% L929 cell-conditioned medium, 10% FBS, and 100 U/ml penicillin–streptomycin for 7 days. The culture medium was changed every two days to obtain BMDMs. Cells were seeded at 1 × 10^6^ cells/ml in 12-well or 6-well plates overnight. The next day, macrophages were stimulated with LPS (0.5 *μ*g/ml) for 4 hours followed by challenge with NLRP3 activators ATP (3 mM) and nigericin (10 *μ*M) for 1 hour or MSU (300 *μ*g/ml) for 6 hours. Cell culture medium and cell lysates were gathered for subsequent experiments.

### 2.4. Cell Death Detection

After stimulation, PI (2 *μ*g/ml) and Hoechst 33342 (5 *μ*g/ml) were loaded onto the cell culture plate and incubated at room temperature for 10 min. Immediately after staining, a BIO-RAD ZOE™ Fluorescent Cell Imager was used to take images of multiple cell fields. Multiple cell fields were randomly selected and photographed to count the frequency (%) of PI-positive cells.

LDH release was detected by LDH cytotoxicity assay kit to analyze cell death. 120 *μ*l cell supernatant and 60 *μ*l prepared LDH reagent were loaded to a 96-well plate, and then incubated on a horizontal shaker in the dark for 30 minutes. A PerkinElmer EnSpire was employed to measure the absorbance at 490 nm.

### 2.5. Enzyme-Linked Immunosorbent Assay (ELISA)

According to the manual of the ELISA manufacturer, mouse IL-1*β* and TNF-*α* ELISA kits were used to determine cytokine by macrophages. In brief, the supernatant or standard sample was loaded to the precoated plate and maintained at 37°C for 90 min. Then the plate was washed with a washing solution five times. Biotinylated antibody working solution was loaded and reacted at 37°C for 1 hour in a dark place. Wash the plate five times. The enzyme conjugate working solution was loaded to the samples and maintained at 37°C for 30 min. Next, chromogenic substrate (TMB) was loaded to the plate and maintained at 37°C for 15 min. Finally, the reaction was blocked by the stop solution. Samples were quantified in a PerkinElmer EnSpire to capture absorbance at 450 nm. The cytokine concentration of each sample was analyzed with ELISACalc software using a standard curve method.

### 2.6. Immunofluorescence and Confocal Microscopy

Macrophage or joint tissue was fixed in 4% paraformaldehyde, followed by permeabilization with 0.5% Triton X-100, and blocked in 1× PBS supplemented with 3% normal goat serum. The samples were maintained in primary antibodies at 4°C overnight, and then combined with secondary alexa antibodies from Cell Signaling Technology for 1 h. Cell nuclei were counterstained with Hoechst 33342. Zeiss LSM 800 confocal laser scanning microscope was employed to capture fluorescent images.

### 2.7. Western Blotting

To concentrate cell supernatants for immunoblotting, 800 *μ*l of cell supernatant, an equal volume of methanol and 1/4 volume of chloroform were mixed and vortexed for 1 min. The mixtures were centrifuged at 12000 rpm at 4°C for 10 min. Supernatants were removed, and the remaining pellet was resuspended in 35 *μ*l 1× sample loading buffer. Macrophages were lysed by RIPA buffer supplemented with the protease and phosphatase inhibitor cocktail. Whole protein density was measured by a BCA Protein Assay Kit. Concentrated supernatants and cell lysates were denatured under 95°C for 5 min. The denatured protein samples were separated by SDS–PAGE and transferred onto the PVDF membrane. The next day, the PVDF membrane was blocked in 5% nonfat milk in 1× TBST at room temperature for 1 h. The samples were maintained in the primary antibodies at 4°C overnight, followed by incubation with the secondary antibodies at room temperature for 1 h. The PVDF membrane was detected using a picogram ECL kit (Affinity, KF001). Protein visualization occurred on a Tanon 4600 imaging system (Tanon, Shanghai, China).

### 2.8. ASC Oligomerization and Speck Formation Assay

Macrophages were treated as described above. Cells were fixed in 4% paraformaldehyde at room temperature for 15 min, followed by permeabilization with ice-cold methanol. Then macrophages were incubated with anti-ASC antibody with a final dilution of 1 : 300 at 4°C overnight and Alexa Fluor 488 goat anti-rabbit IgG antibody for 1 h. Cell nuclei were counterstained with Hoechst 33342. ASC specks images were captured under a Zeiss Axio observer 3 fluorescence microscope.

LPS-primed BMDMs were treated with ATP or nigericin as stated above. Then, macrophages were lysed in ice-cold PBS supplemented with 0.5% Triton-X 100 and a protease inhibitor cocktail for 30 min. Cell lysates were centrifuged at 6,000 × g at 4°C for 15 min. The remaining pellets were washed twice with PBS and resuspended in 200 *μ*l PBS. Disuccinimidyl suberate (2 mM) was loaded onto the pellets and maintained at room temperature for 30 min to cross-link. The samples were centrifuged at 6,000 × g at 4°C for 15 min. 30 *μ*l loading buffer was added to the cross-linked pellets and subsequently boiled for 5 min. The sample was run on a gel and immunoblotted using an anti-ASC antibody.

### 2.9. Coimmunoprecipitation (Co-IP)

BMDMs were lysed in ice-cold cell lysis buffer for western blotting and IP. Cell lysates were transferred into microcentrifuge tubes and centrifuged at 12000 rpm at 4°C for 15 min. The protein concentration of each sample was determined using a BCA Protein Assay Kit. Cell lysate samples were dispensed and precleared on Protein A/G agarose beads with mild rotation at 4°C for 30 min. The sample was maintained in anti-TXNIP antibody or IgG antibody on a rotational cell mixer at 4°C overnight. The next day, protein A/G agarose beads were added to the immune complexes and gently rotated at 4°C for 2 h. The samples were then centrifuged. The beads were washed with cell lysis buffer five times. The proteins of each sample were eluted from washed beads by boiling for 5 min in 3× SDS loading buffer and analyzed by SDS–PAGE.

### 2.10. MitoSOX and MitoTracker Staining

LPS-primed macrophages were stimulated with the NLRP3 inflammasome activators. MitoSOX was used to detect mitochondrial ROS (mtROS). MitoTracker was employed to assess mitochondrial morphology. MitoTracker (200 nM) or MitoSOX red (5 *μ*M) was loaded and incubated for 30 min before the stimulation was complete. BMDMs were washed with PBS three times and counterstained with Hoechst 33342. Cells' fluorescent images were observed and took photographed through a Zeiss LSM 800 confocal laser scanning microscope.

### 2.11. Mitochondrial Morphology Analysis

Open Fiji-ImageJ (NIH) and click Plugins to install MiNA macros. Images from the Zeiss LSM 800 were loaded into FIJI for mitochondria morphology analysis. The binary image was converted into a skeleton representing the features in the original image by using “skeletonize”. Next, the mean branch length and the number of individuals were determined by using the “Analysis Skeleton” plug-in.

### 2.12. Mitochondrial Membrane Potential Assay with JC-1 Kit

After treating the cells as described above, aspirate the old medium. Cells were loaded with JC-1 working solution and maintained in a cell incubator at 37°C for 20 minutes in the dark. Aspirate the supernatant and wash with JC-1 staining buffer twice. The mitochondrial membrane potential is high in healthy cells, and JC-1 exists as aggregates with bright red fluorescence. When the mitochondrial membrane potential decreased, the number of JC-1 monomers increased and showed green fluorescence. Then the cell fluorescence images were observed and captured using a laser scanning microscope.

### 2.13. MSU-Induced Gouty Arthritis in Mice

A knee joint gouty arthritis mice model was established according to our previous studies [16, 17]. Corilagin (20 mg/kg) or colchicine (1 mg/kg) was intraperitoneally injected into the right knee joint of C57BL/6 J mice colchicine treated group was a positive control. After one hour, 0.5 mg MSU crystals resuspended in 20 *μ*l of sterile PBS were injected into the knee joint of each mouse. We determined the joint diameter using an electronic caliper at the indicated time of up to 24 hours. The knee joints were isolated and incubated in Opti-MEM supplemented with 100 U/ml penicillin–streptomycin for 1 h. The joint culture medium was collected for cytokine measurement. The harvested knee joint was fixed and decalcified for histological staining.

### 2.14. Histological Evaluation and Immunohistochemistry

The knee joint was embedded in paraffin. Then the joint tissue was sectioned and stained with hematoxylin and eosin (H&E) to determine morphological injury and inflammation. In addition, joint sections were deparaffinized, rehydrated, and unmasked with antigen in citrate buffer at 60°C overnight. The joint sections were incubated with 3% hydrogen peroxide, followed by permeabilization with 0.1% Triton X-100 in PBS. After washing, the joint sections were maintained in 3% sheep serum at room temperature for 1 h. The joint sections were incubated with an antibody specific for F4/80 (macrophage marker) or myeloperoxidase (Ly-6G, neutrophil marker). NLRP3 inflammasome components of the joint were immunostained with caspase-1 p20 or IL-1*β* antibodies at 4°C overnight. Joint sections were maintained in HRP-conjugated secondary antibody and then reacted with DAB. Then the cell nuclei were counterstained with hematoxylin. Immunohistochemistry images were taken pictures by a Zeiss Axio observer 3 microscope. Moreover, the joint sections were maintained in alexa secondary antibody at 37°C for 1 h. Hoechst 33342 was used to stain cell nuclei. The fluorescence pictures were captured through a Zeiss LSM 800 confocal laser scanning microscope.

### 2.15. Statistical Analysis

The data are represented as the mean ± standard deviation (SD) or mean ± standard error of the mean (SEM). The statistical analysis method and the significance levels are also displayed in the respective figure legends. Either Student's *t*-test (two-tailed, unpaired) or one-way ANOVA was employed to analyze raw data, followed by either Tukey's or Dunnett's correction test using GraphPad Prism 8 (GraphPad Software Inc, La Jolla, CA). *P* value less than 0.05 was considered significant.

## 3. Results

### 3.1. Corilagin Blocks NLRP3 Inflammasome Activation and Pyroptosis

Corilagin exerts anti-inflammatory activity mainly by abating proinflammatory cytokines expression, but the mechanism is uncertain. NLRP3 inflammasome activation and pyroptosis contribute to promoting the maturation of inflammatory cytokines such as IL-1*β*. LPS-primed BMDMs were stimulated with classical NLRP3 inflammasome activators (ATP and nigericin) in the presence of corilagin. The proportion of cell death was analyzed by propidium iodide (PI) staining and LDH release detection. ELISA determined the density of IL-1*β* in culture supernatants. In addition, immunoblotting was performed to detect caspase-1 p20 (20 kDa) and mature IL-1*β* (17 kDa) in the supernatant to identify inflammasome activation and the expression of the NLRP3 inflammasome component and pyroptosis executive protein GSDMD in the cell lysate.

**Figure 1:**
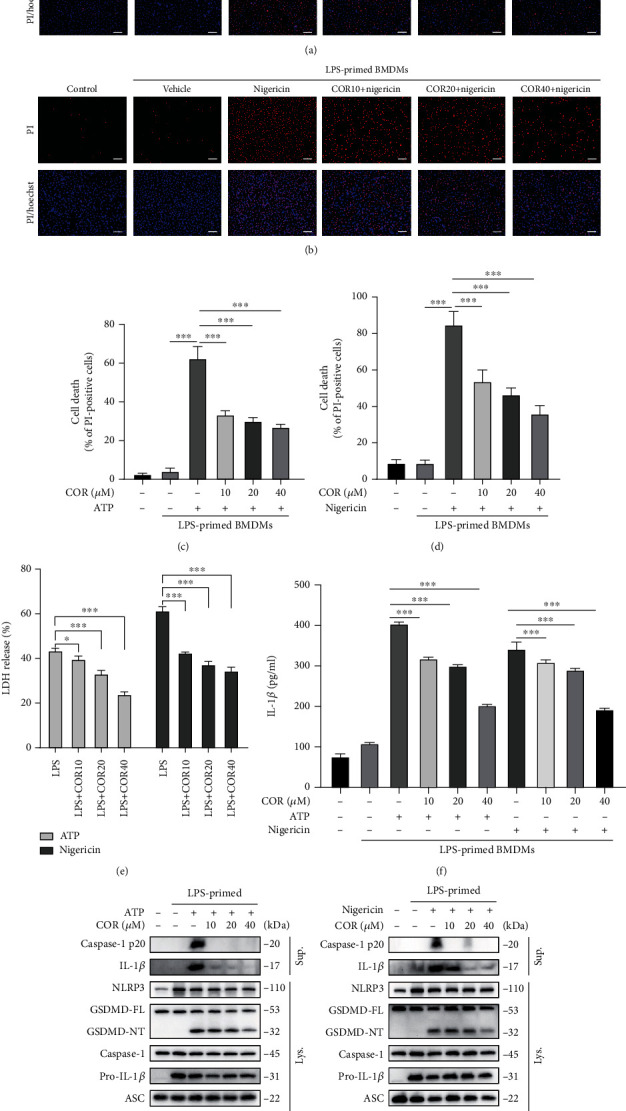
Corilagin inhibits NLRP3 inflammasome activation and pyroptosis in BMDMs. (a–h) BMDMs were primed with LPS (0.5 *μ*g/ml) for 4 h and then stimulated with ATP (3 mM) or nigericin (10 *μ*M) for 1 h with or without corilagin. (a–d) Cells were stained with PI (2 *μ*g/ml) and Hoechst 33342 (5 *μ*g/ml) for 10 min, and the percentage of PI-positive (red) cells relative to total cells (Hoechst 33342, blue) was analyzed (*n* = 6). Scale bar, 100 *μ*m. (e) Cell supernatant was collected to detect LDH release. (f) ELISA determined IL-1*β* level in supernatants. (g and h) Western blotting was used to detect protein expression in the culture supernatant (Sup.) and cell lysate (Lys.). COR: corilagin. ^∗^*P* < 0.05, ^∗∗^*P* < 0.01, ^∗∗∗^*P* < 0.001.

The results showed that corilagin dose-dependently reduced ATP- and nigericin-caused mortality in LPS-primed BMDMs (Figures 1(a)–1(d)) and limited LDH and IL-1*β* release in the supernatant (Figures 1(e) and 1(f)). Western blot results showed that LPS stimulated NLRP3 and pro-IL-1*β* expression, whereas corilagin did not influence NLRP3, pro-IL-1*β*, ASC, or caspase-1 expression in LPS-primed and NLRP3-activated cells. Corilagin significantly inhibited ATP- and nigericin-induced release of caspase-1 p20 and mature IL-1*β* (17 kDa) into the culture supernatant and decreased GSDMD-NT expression (Figures 1(g) and 1(h)). LPS binds to TLR4 to activate the NF-*κ*B pathway and gene transcription. BMDMs were pretreated with corilagin before LPS stimulation, followed by challenge with or without nigericin. The immunoblotting results showed that corilagin did not inhibit LPS-activated phospho (p)-P65 and p-I*κ*B and could not abolish LPS-induced I*κ*B degradation (Figure S1). The result indicated that corilagin could not inhibit TLR4/NF-*κ*B pathway. Collectively, we conclude that corilagin specifically obstructs NLRP3 inflammasome activation and macrophage pyroptosis, thereby reducing IL-1*β* release.

### 3.2. Corilagin Suppresses ASC Oligomerization and Speck Formation

**Figure 2:**
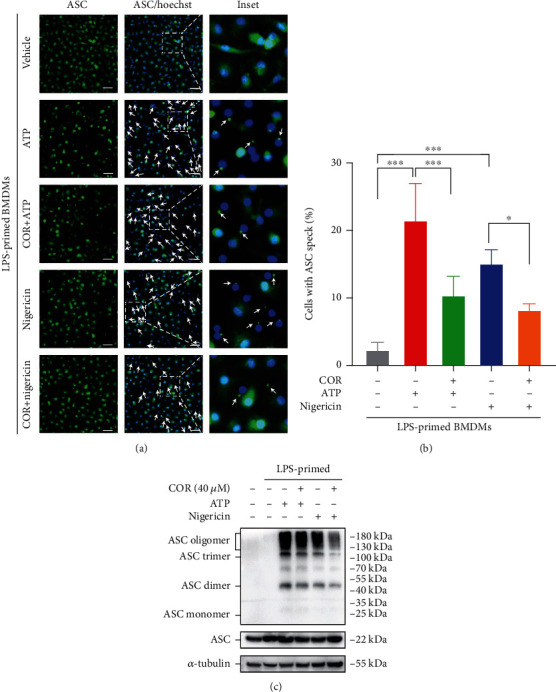
Corilagin prevents ASC aggregation in BMDMs. (a–c) BMDMs were primed with LPS (0.5 *μ*g/ml) for 4 hours and then incubated with ATP (3 mM) or nigericin (10 *μ*M) for 1 hour with or without corilagin (40 *μ*M). (a) Representative image of cell immunofluorescence stained for ASC (green). Scale bar, 20 *μ*m. (b) Percentages of cells shown in (a) with ASC specks (*n* = 5). (c) Western blotting was applied to analyze ASC oligomerization in ATP- or nigericin-treated BMDMs. *α*-Tubulin is the loading control. COR: corilagin. ^∗^*P* < 0.05, ^∗∗∗^*P* < 0.001.

NLRP3 inflammasome is a multiprotein complex. ASC acts as a linker between NLRP3 and caspase-1 and participates in caspase-1 activation. NLRP3 activation is accompanied by ASC protein oligomerization, and ASC specks are a significant marker of NLRP3 inflammasome aggregation. ATP and nigericin were added to stimulate LPS-primed macrophages for NLRP3 inflammasome activation. Immunofluorescence results displayed that ASC specks were increased and irregularly distributed in the positive group, and corilagin significantly blocked ASC speck formation (Figures 2(a) and 2(b)). Furthermore, ASC oligomerization was detected by chemical cross-linking and western blotting. The results showed that corilagin inhibited ATP- and nigericin-caused ASC oligomerization in LPS-stimulated BMDMs (Figure 2(c)). Altogether, these results indicate that corilagin can reduce ASC speck formation by blocking ASC oligomerization, which reveals its potential to restrict NLRP3 inflammasome assembly and activation.

### 3.3. Corilagin Prevents Inflammasome Activation Dependent on NLRP3

**Figure 3:**
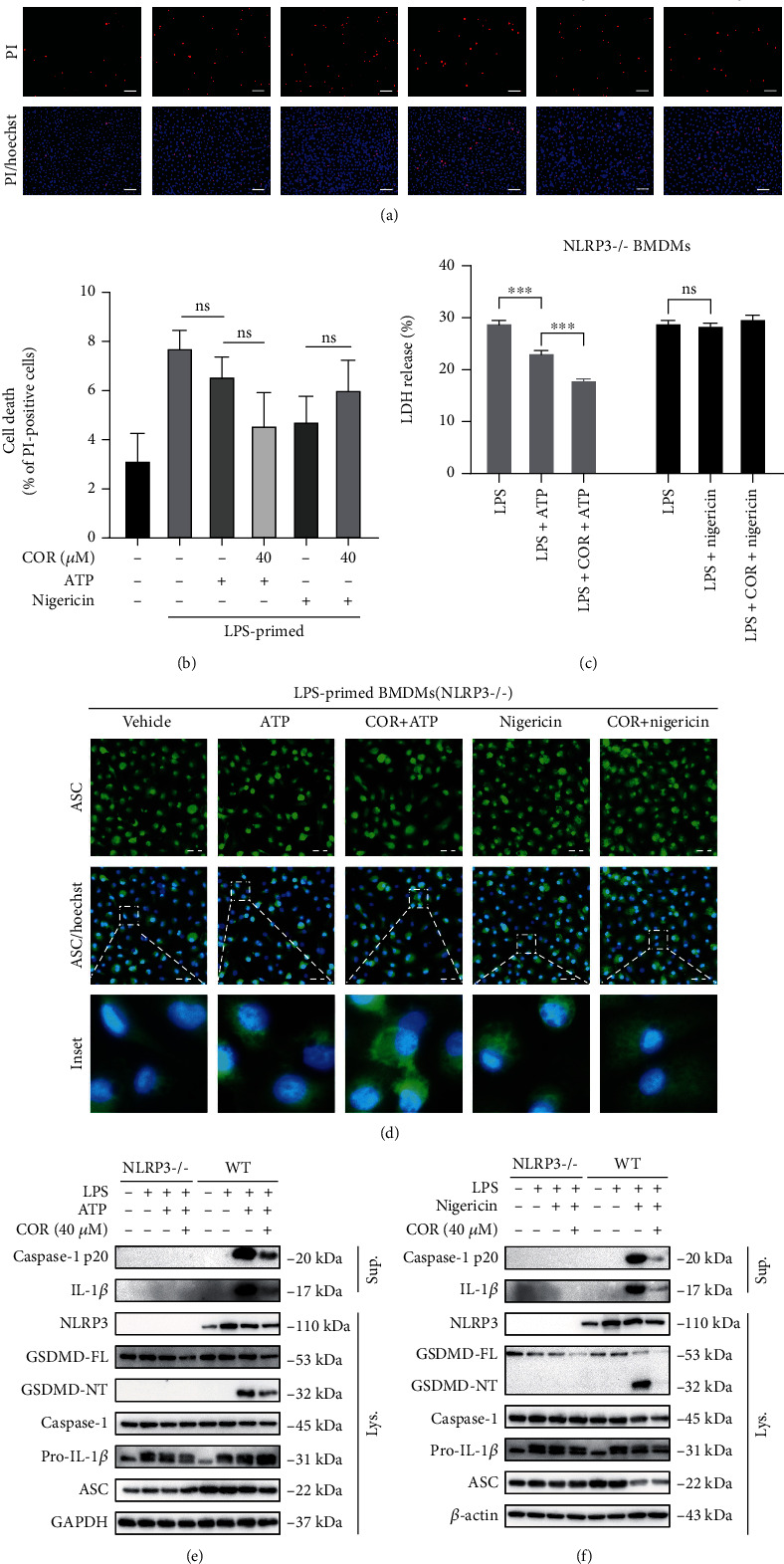
Corilagin restrains inflammasome activation and pyroptosis in an NLRP3-dependent manner. (a–d) NLRP3^−/−^ BMDMs and WT BMDMs were pretreated with LPS (0.5 *μ*g/ml) for 4 hours and then stimulated with ATP (3 mM) or nigericin (10 *μ*M) in the presence of corilagin for 1 hour. (a) NLRP3^−/−^ BMDMs were stained with PI and Hoechst 33342 for 10 min. Scale bar, 100 *μ*m. (b) The percentage of PI-positive (red) cells relative to total cells (Hoechst 33342, blue) was analyzed (*n* = 5). (c) The LDH release in the supernatant was detected. (d) The distribution of ASC in BMDMs was observed by immunofluorescence. Scale bar, 20 *μ*m. (e and f) Western blotting was used to analyze the proteins in the supernatant concentrate and cell lysate. COR: corilagin; WT: wild type. ^∗∗∗^*P* < 0.001.

To further prove the specificity of corilagin in restricting the NLRP3 inflammasome, bone marrow cells from NLRP3-deficient (NLRP3^−/−^) mice and wild-type (WT) mice were isolated and induced to obtain BMDMs. NLRP3 inflammasome activation was then induced with LPS and ATP or nigericin. PI staining results displayed that few cells died in NLRP3^−/−^ BMDMs, and corilagin showed no noticeable antipyroptosis effect (Figures 3(a) and 3(b)). LDH release was inhibited by corilagin in ATP-activated cells but not nigericin-stimulated cells (Figure 3(c)). In addition, ASC specks were not observed in NLRP3^−/−^ BMDMs (Figure 3(d)). ATP and nigericin could not trigger the generation of caspase-1 p20, mature IL-1*β*, and GSDMD-NT (Figures 3(e) and 3(f)). Almost no secreted IL-1*β* was detected in the supernatant of NLRP3^−/−^ BMDMs (Figure S2). In summary, these results demonstrate that corilagin prevents NLRP3-dependent inflammasome activation and pyroptosis.

### 3.4. Corilagin Diminishes ROS Generation and the Interaction between TXNIP and NLRP3

**Figure 4:**
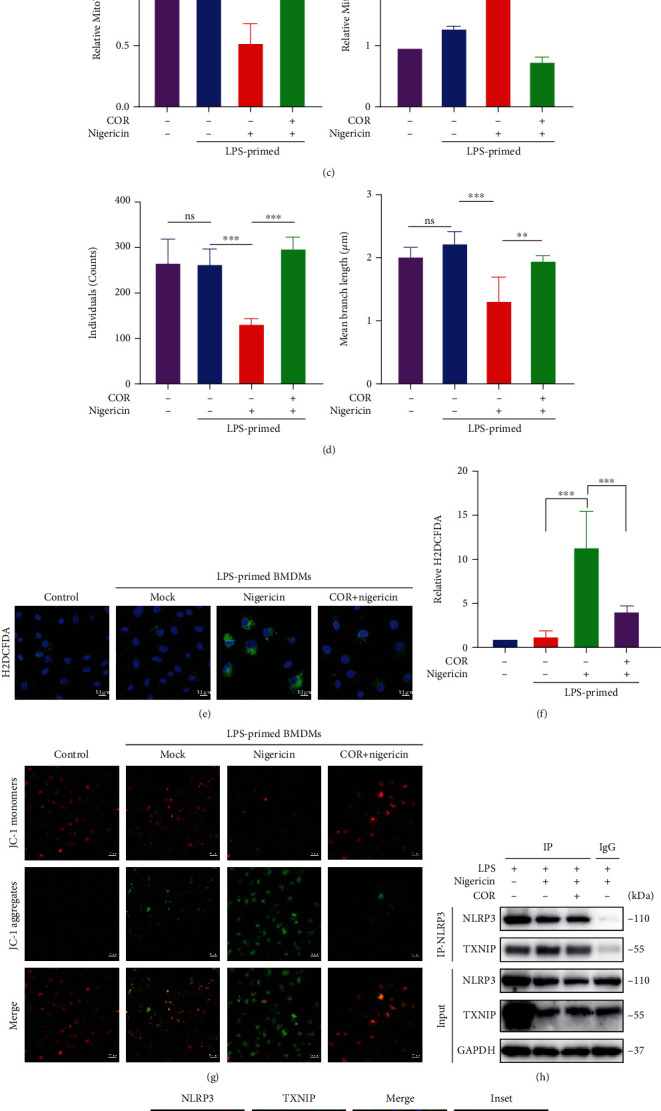
Corilagin decreases the production of mtROS and the NLRP3-TXNIP interaction. (a–i) LPS-primed BMDMs were incubated with or without corilagin (40 *μ*M) for 30 min, and then stimulated with nigericin for 1 h. (a–c) MitoTracker or MitoSOX reagent stained cells for 30 min before the stimulation was complete. A confocal microscope randomly photographed multiple cell regions. (c) Quantification of representative images shown in (a and b). The quantitative statistics of mitochondrial individuals and mean branch length in (a) were represented in (d) (*n* =5). (e) Cellular ROS level was detected by H2DCFDA, and the relative fluorescence intensity was shown in (f). Scale bar, 10 *μ*m. (g) Cells stained with JC-1 and captured by confocal microscopy. Scale bar, 20 *μ*m. (h) Coimmunoprecipitation analysis of the interaction between TXNIP and NLRP3. (i) Representative fluorescent images of BMDM costained for TXNIP and NLRP3. Scale bar, 10 *μ*m. COR: corilagin. ^∗∗∗^*P* < 0.001.

Mitochondrial morphology changes cause increasing mitochondrial reactive oxygen species (mtROS). Excessive ROS induces TXNIP binding to NLRP3 and activates NLRP3 inflammasome [11]. To explore the mechanism of how corilagin inhibited NLRP3 inflammasome activation, we employed MitoTracker to identify mitochondria and MitoSOX Red to stain mtROS. Furthermore, H2DCFDA and JC-1 were used to detect intracellular ROS and mitochondrial membrane potential, respectively. Next, the interaction between TXNIP and NLRP3 was determined by Co-IP assay and immunofluorescence. Our results showed that nigericin significantly induced a fragmented mitochondrial morphology as indicated by a decrease in mitochondrial individuals and mean branch length, while corilagin restored mitochondrial morphology phenotype (Figures 4(a) and 4(d)). Consistently, corilagin reduced the production of mtROS (Figures 4(b) and 4(c)) and intracellular ROS (Figures 4(e) and 4(f)). Besides, corilagin prevented the loss of mitochondrial membrane potential in nigericin-stimulated BMDMs (Figure 4(g)). The study has proved that serine/threonine kinase NEK7 binds to NLRP3 and promotes NLRP3 inflammasome activation [18]. However, the Co-IP results showed that corilagin did not affect the interaction between NEK7 and NLRP3 (Figure S3). Furthermore, we examined the interaction between TXNIP and NLRP3 by immunofluorescence and Co-IP. As shown in Figured 4(h) and 4(i), nigericin caused the colocalization of TXNIP and NLRP3, but corilagin abolished the interaction between TXNIP and NLRP3.

**Figure 5:**
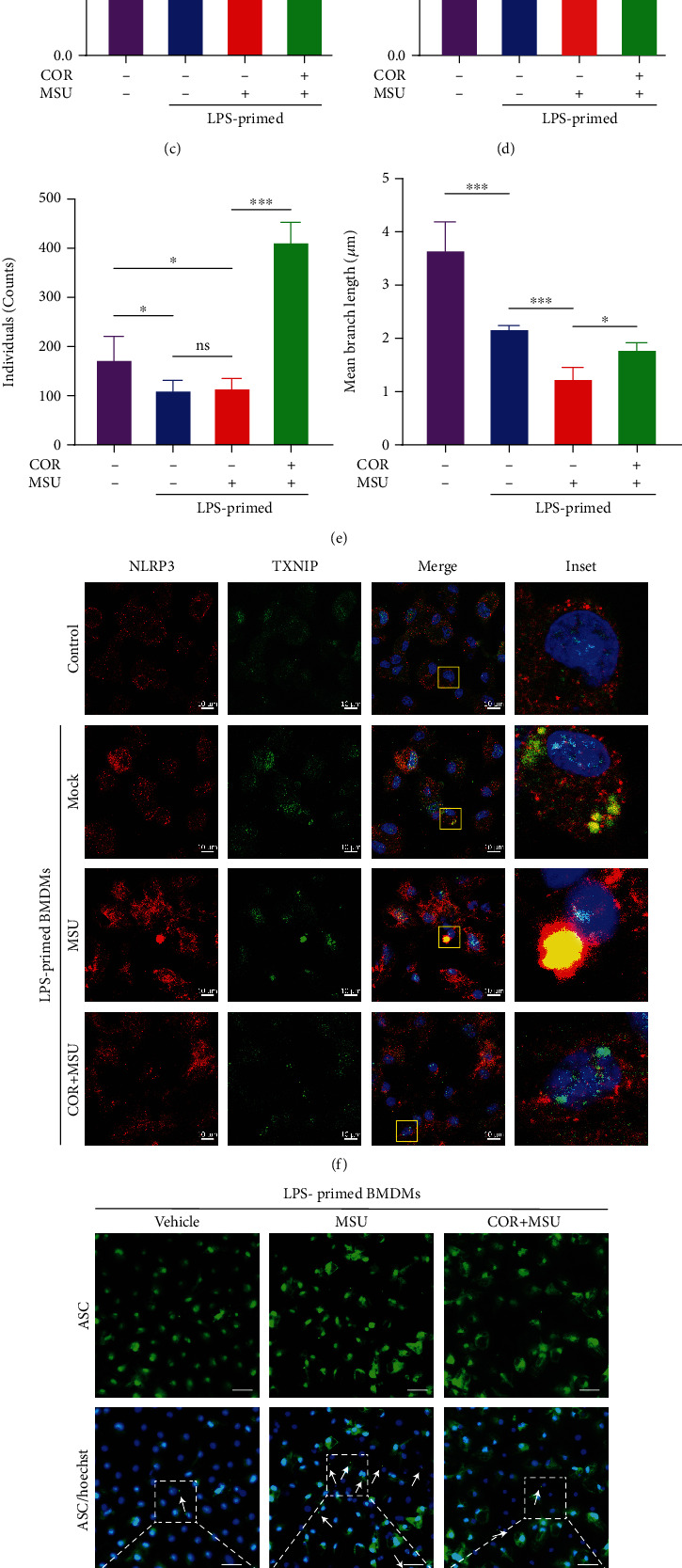
Corilagin inhibits MSU-induced activation of the ROS-TXNIP-NLRP3 pathway. (a–h) LPS-primed BMDMs were treated with corilagin (40 *μΜ*) for 30 min followed by stimulation with MSU (300 *μ*g/ml) for 6 h. (a and b) The cells were stained with MitoTracker or MitoSOX for 30 min, and fluorescence images were taken by the confocal microscope. Statistical analysis of cell fluorescence intensity was shown in (c and d) (*n* = 5). (e) Histograms show the mitochondrial individuals and mean branch length in (a). (f) Immunofluorescence staining of TXNIP (green) and NLRP3 (red). Scale bar, 10 *μ*m. (g) Immunofluorescence analysis of the subcellular distribution of ASC specks (white arrow indicator). Scale bar, 20 *μ*m. Quantitative analysis of the percentage of ASC specks displayed in (h). (i) NLRP3 inflammasome components were analyzed by western blot. COR: corilagin. ^∗∗^*P* < 0.01, ^∗∗∗^*P* < 0.001.

To further identify whether corilagin modulates inflammasome by the ROS-TXNIP-NLRP3 pathway in MSU-stimulated BMDMs. Immunofluorescence results also showed that corilagin inhibited MSU-induced mitochondria fragmentation to restore mitochondrial morphology phenotype (Figures 5(a), 5(c), and 5(e)) and reduced MSU-stimulated mtROS generation (Figures 5(b) and 5(d)) and NLRP3-TXNIP interaction (Figure 5(f)). In addition, corilagin prevented MSU-induced ASC speck formation (Figures 5(g) and 5(h)) and decreased the production of caspase-1 p20 and mature IL-1*β* in the culture medium (Figure 5(i)). Collectively, these results demonstrate that corilagin restrains TXNIP-NLRP3 interaction by inhibiting mitochondrial morphology changes and mtROS generation to prevent NLRP3 inflammasome activation.

### 3.5. Corilagin Inhibits Inflammasome Activation and Pyroptosis Depending on the ROS/TXNIP/NLRP3 Pathway

A variety of molecules regulate the NLRP3 inflammasome, in which the PKA signal inhibits the inflammasome by increasing the phosphorylation of NLRP3 at Ser/Thr residues [19, 20]. Nrf2 blocks NLRP3 inflammasome activation by promoting antioxidant activity [21]. We adopted the PKA inhibitor H89 and the Nrf2 inhibitor ML385, and the results showed that these two inhibitors did not counteract the effect of corilagin (Figure S4). Imiquimod can induce decreased mitochondrial respiratory activity and dynamic imbalance [22]. Imiquimod can activate the NLRP3 inflammasome by inducing ROS generation [12]. Since corilagin is a potential antioxidant, we employed imiquimod to determine whether corilagin inhibits the NLRP3 inflammasome by modulating ROS production.

**Figure 6:**
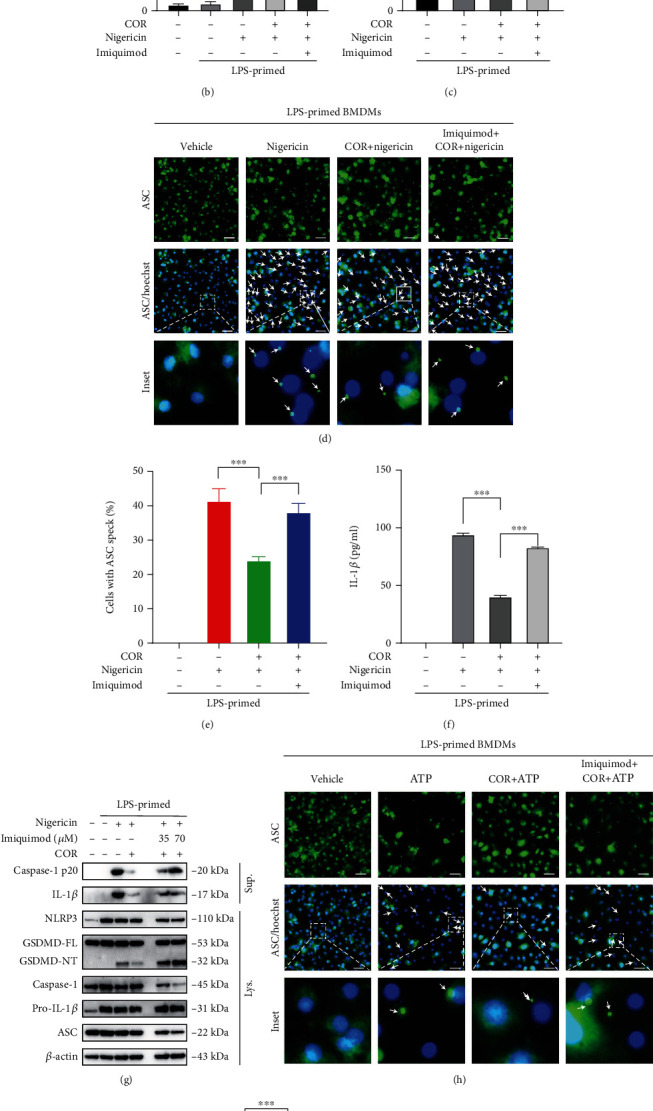
Imiquimod resists the inhibitory effect of corilagin on NLRP3 inflammasome activation and pyroptosis. (a–f, h and i) LPS-primed BMDMs were incubated with imiquimod (70 *μ*M) for 30 min, treated with corilagin (40 *μ*M) for 30 min, and then stimulated with nigericin or ATP for 1 h. (a) Representative images of cell death (PI positive) are shown in BMDMs staining with Hoechst 33342 and PI. Scale bar, 100 *μ*m. (b) Quantification of cell death in (a). (c) Analysis of culture supernatant levels of LDH. (d and h) Representative immunofluorescence images of BMDMs stained for ASC. White arrows indicate ASC speck. Scale bar, 20 *μ*m. (e and i) Quantification of the ASC specks in (d and h) (*n* = 6). (f) IL-1*β* level was assayed by ELISA kit. (g and j) Immunoblot analysis of pro- and activated (p20) caspase-1, pro- and mature IL-1*β*, GSDMD, NLRP3, and ASC. *β*-Actin was used as the internal control.

Our data showed that imiquimod reversed the effect of corilagin in decreasing pyroptosis and LDH release in nigericin-stimulated BMDMs (Figures 6(a)–6(c)). Additionally, corilagin reduced ASC specks in ATP- or nigericin-stimulated BMDMs, while imiquimod restored ASC speck formation (Figures 6(d), 6(e), 6(h), and 6(i)). Imiquimod also restored IL-1*β* expression upon corilagin incubation in LPS- and nigericin-treated cells (Figure 6(f)). Moreover, corilagin restrained caspase-1 p20, mature IL-1*β*, and GSDMD-NT production, while imiquimod antagonized the function of corilagin. (Figures 6(g) and 6(j)). Overall, we conclude that imiquimod resists the inhibitory function of corilagin on NLRP3 inflammasome activation and macrophage pyroptosis.

**Figure 7:**
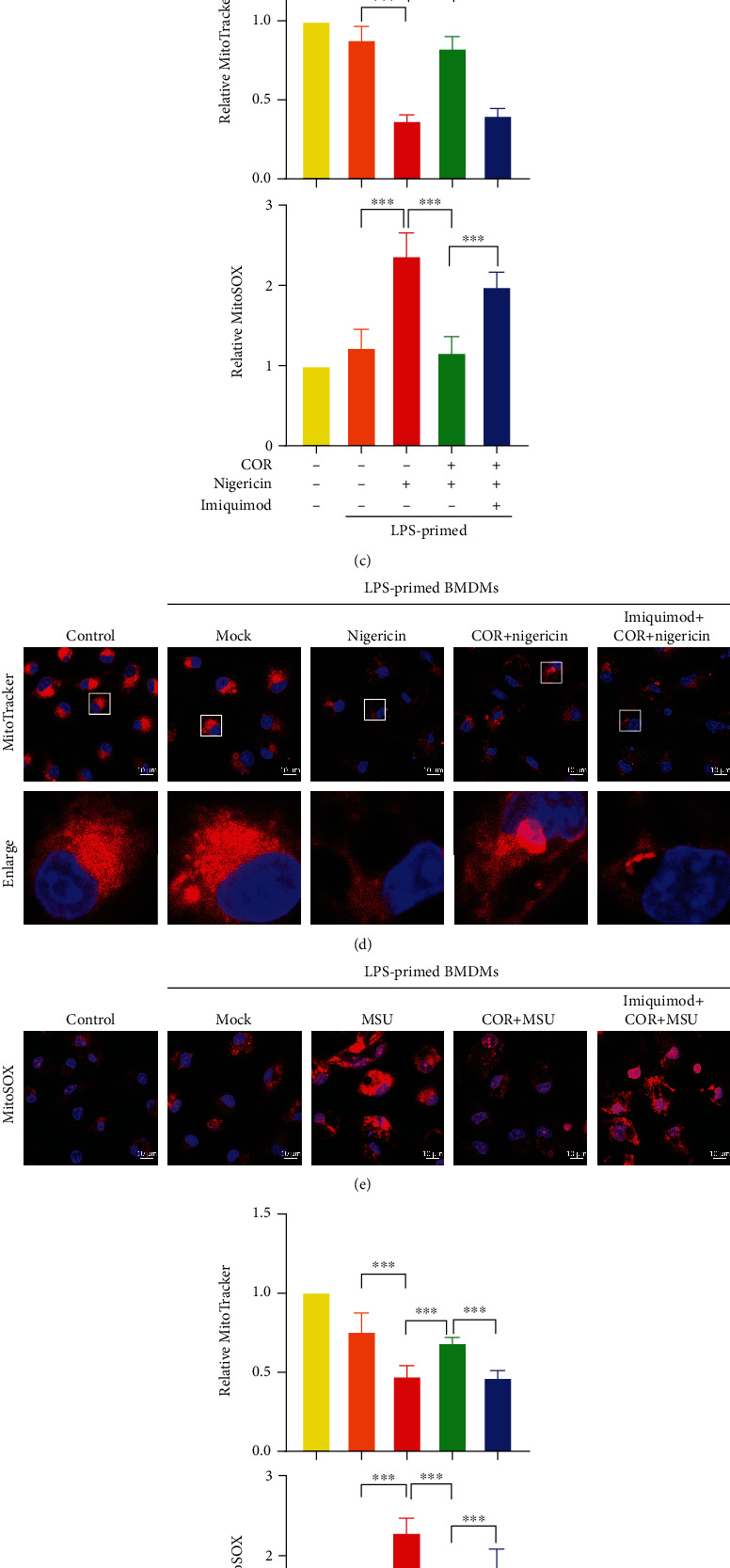
Imiquimod counteracts the effect of corilagin by activating the ROS/TXNIP/NLRP3 pathway. (a–g) LPS-primed BMDMs were treated with corilagin (40 *μ*M) in the presence or absence of imiquimod (70 *μ*M), and then stimulated with MSU. (a and b, d and e) BMDMs were stained with MitoTracker or MitoSOX for 30 min. Fluorescence images were taken by the confocal laser scanning microscope. Scale bar, 10 *μ*m. (c and f) The relative fluorescence intensity of MitoTracker or MitoSOX was analyzed and normalized to the control (*n* = 5). (g) Immunoprecipitation analysis of the interaction between TXNIP and NLRP3. COR: corilagin. ^∗∗∗^*P* < 0.001.

Imiquimod can significantly increase the production of mitochondrial ROS [23]. However, whether imiquimod neutralizes the effect of corilagin via the mitochondrial ROS pathway remains unknown. Upon nigericin or MSU stimulation, LPS-primed macrophages were incubated with or without imiquimod, followed by treated with corilagin. MitoTracker fluorescence images showed that corilagin protected macrophages from nigericin- and MSU-induced mitochondrial morphology changes, while imiquimod antagonized the effect of corilagin and exacerbated mitochondrial morphology fragmentation (Figures 7(a), 7(c), 7(d), and 7(f)). MitoSOX fluorescence images showed that corilagin could reduce mtROS levels, while imiquimod enhanced mtROS generation in the presence of corilagin (Figures 7(b), 7(c), 7(e), and 7(f)). Immunoprecipitation results revealed that corilagin inhibited MSU-induced TXNIP binding to NLRP3, while imiquimod promoted the TXNIP-NLRP3 interaction (Figure 7(g)). These results demonstrate that imiquimod abrogates the effect of corilagin on reducing mitochondrial morphology changes and mtROS generation, further confirming that corilagin inhibits inflammasome activation partly through the ROS/TXNIP/NLRP3 pathway.

### 3.6. Corilagin Ameliorates Monosodium Urate Crystals-Induced Gouty Arthritis

**Figure 8:**
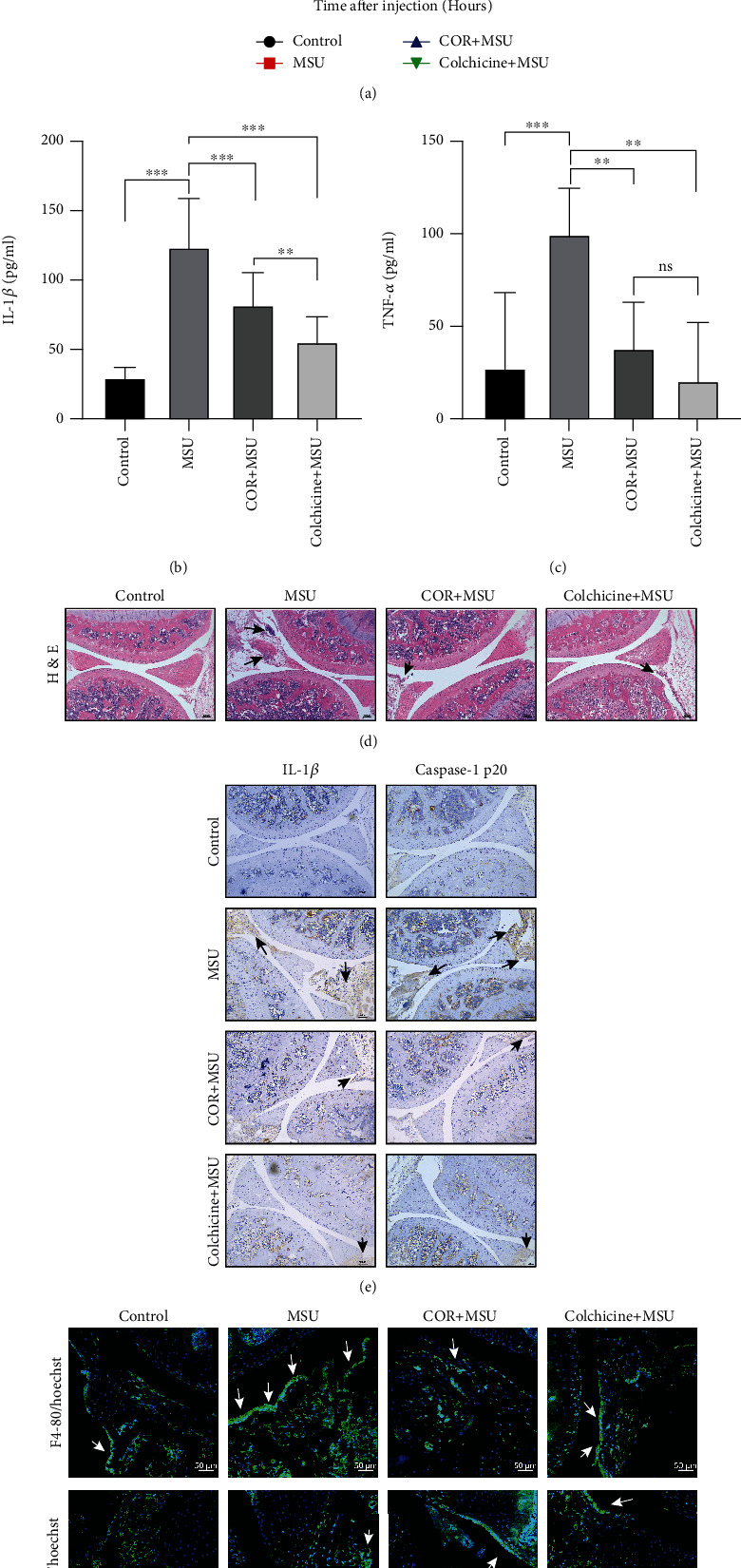
Corilagin relieves MSU-induced arthritis in C57BL/6 J mice. (a–g) C57BL/6 J mice were intraperitoneal injection with corilagin (20 mg/kg) or colchicine (1 mg/kg), and then MSU crystals were injected into the knee joint for 24 h. (a) Joint swelling was measured at different times. (b and c) Levels of IL-1*β* and TNF-*α* were detected by ELISA kits (*n* = 6). (d) H&E staining of knee joint section. Black arrows indicate cell infiltration. Scale bar, 100 *μ*m. (e) Immunohistochemistry analysis for IL-1*β* and caspase-1 p20. Scale bar, 100 *μ*m. (f) Immunofluorescence staining of F4/80 and Ly-6G in joint sections. Scale bar, 50 *μ*m. ^∗∗∗^*P* < 0.001.

MSU crystals can motivate the activation of NLRP3 inflammasome, which triggers the gout flare. We have demonstrated that corilagin can effectively inhibit MSU-stimulated NLRP3 inflammasome activation *in vitro*. Therefore, MSU crystals-caused gouty arthritis were employed to explore the anti-inflammation activity of corilagin *in vivo,* and colchicine was taken as the positive control. The results reveal that corilagin alleviates MSU crystals-caused knee swelling in mice (Figure 8(a)). ELISA was applied to detect inflammatory cytokines in the culture supernatant of the isolated knee joint. We found that both corilagin and colchicine could reduce IL-1*β* and TNF-*α* generation (Figures 8(b) and 8(c)). We also observed that corilagin and colchicine restrained MSU-induced inflammatory cell infiltration (Figure 8(d)). Immunohistochemical results showed that corilagin and colchicine decreased the production of IL-1*β* and caspase-1 p20 in the joint tissue (Figure 8(e)).

To further determine which inflammatory cells gather, the joint tissue was stained with Ly-6G (neutrophil marker) antibody or F4/80 (macrophage marker) antibody. The immunofluorescence image results showed that MSU attracted the neutrophils and macrophages aggregation in the joint, but corilagin and colchicine reduced the accumulation of these inflammatory cells (Figure 8(f)). Collectively, we conclude that corilagin alleviates MSU crystals-caused gouty arthritis by restraining NLRP3 inflammasome activation, inflammatory cytokines expression, and immune cell infiltration.

## 4. Discussion

Colchicine, NSAIDs, or glucocorticoids are often used as first-line drugs to treat gout flares, but several patients have poor tolerance or contraindications to these common anti-inflammatory therapies. Here, we find that corilagin, a compound derived from traditional Chinese medicine, alleviates MSU-induced acute gouty arthritis by preventing NLRP3 inflammasome and reducing IL-1*β* production and immune cell infiltration, and no significant adverse reactions were observed. We provide evidence that corilagin inhibits the interaction between TXNIP and NLRP3 by reducing mitochondrial morphology changes and ROS generation. Ultimately, corilagin suppresses NLRP3 inflammasome activation and the subsequent release of mature IL-1*β*.

Corilagin has been demonstrated to prevent inflammation by inhibiting the production of proinflammatory cytokines such as IL-1*β* and TNF-*α* [24]. In addition, corilagin exerts its potent antioxidant effect by decreasing ROS expression [25–27]. Consistent with the previous studies, we find that corilagin significantly prevents inflammasome activation and IL-1*β* expression (Figures 1, 2, and 5), and this effect depended on NLRP3 (Figure 3). Excessive mitochondrial ROS promotes TXNIP binding to NLRP3 and thus induces NLRP3 inflammasome activation [10, 11]. We show herein that corilagin limits mitochondria morphology changes, reduces mtROS generation, and prevents the interaction between TXNIP and NLRP3. (Figures 4 and 5). These findings suggest that corilagin suppresses NLRP3 inflammasome activation through the ROS/TXNIP/NLRP3 pathway. Lian et al. also confirmed that ROS/TXNIP/NLRP3 is a critical priming signaling pathway for periodontitis [28]. Metformin has been shown to inhibit TXNIP expression and the interaction between TXNIP and NLRP3 and decrease caspase-1 and GSDMD-NT expression, thereby protecting barrier function from intestinal ischemia-reperfusion injury and abating oxidative stress and inflammation [29]. These results reveal that targeting the ROS/TXNIP/NLRP3 pathway could be a potential anti-inflammation strategy. Imiquimod, a ROS activator, can reverse the inhibitory function of corilagin on the NLRP3 inflammasome and macrophage pyroptosis (Figure 6). Furthermore, imiquimod promotes the binding of TXNIP to NLRP3 by inducing mitochondrial morphology changes and ROS production (Figure 7). Our results preliminarily proved that corilagin limited NLRP3 inflammasome activation and the macrophage pyroptosis through antioxidant effects.

Multiple molecules and pathways regulate the NLRP3 inflammasome. NEK7, a serine/threonine kinase is an integral part of the NLRP3 inflammasome. NEK7 binds to the inflammasome by binding to the LRR domain of NLRP3, which is necessary for the activation of the NLRP3 inflammasome [18]. However, the Co-IP analysis results revealed that corilagin did not prevent the interaction between NEK7 and NLRP3 (Figure S3). In addition, our previous studies have proved that wedelolactone increases Ser/Thr phosphorylation of NLRP3 by enhancing the PKA signal, thereby impeding NLRP3 inflammasome activation and macrophage pyroptosis. Meanwhile, the PKA inhibitor H89 can reverse the function of wedelolactone [16]. Gallic acid enhances Nrf2 signaling and reduces mtROS production to limit NLRP3 inflammasome activation, while the Nrf2 inhibitor ML385 antagonizes the effect of gallic acid [17]. Cells were treated with H89 and ML385 during stimulation. However, these two inhibitors could not counteract the effect of corilagin (Figure S4). The results conclude that corilagin obstructs NLRP3 inflammasome independent of the PKA or Nrf2 pathway.

Colchicine, a microtubule-depolymerizing agent used in the prevention and treatment of acute gout attacks, reduces the MSU crystals-induced painful inflammatory response in joints [13]. However, colchicine often causes gastrointestinal and other adverse reactions. The *in vivo* results suggest that the gastrointestinal reactions of corilagin are significantly lower than in colchicine treated group. Corilagin reduces immune cell infiltration and inflammatory symptoms in MSU-induced acute gouty arthritis (Figure 8). Corilagin mainly inhibits the infiltration of macrophages and neutrophils by blocking NLRP3 activation and IL-1*β* release. Although corilagin treats gouty arthritis by obstructing the NLRP3 inflammasome, the *in vivo* function of corilagin in mediating the ROS/TXNIP/NLRP3 pathway remains unclear. In addition, it is also unclear whether corilagin can lower serum uric acid. Corilagin displays extensive pharmacological activities in cells and mice models. One study indicated that corilagin (100 *μ*M) displayed no cytotoxicity to human normal cells (LO2, BEAS-2B, and HEK293). Moreover, oral administration of corilagin (300 mg/kg) showed no apparent toxicity or detrimental effects, mice body weight and organ weight also did not change significantly [30]. However, there are fewer clinical trials of corilagin *in vivo*. Therefore, the safety and clinical transformation of corilagin in humans deserve further study.

**Figure 9:**
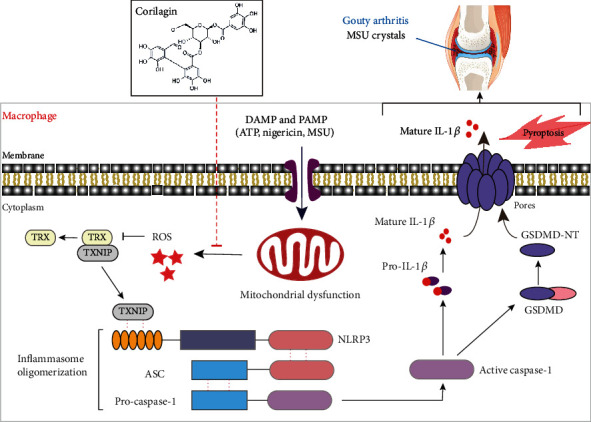
The proposed mechanism. Corilagin restrains NLRP3 inflammasome activation and IL-1*β* secretion to prevent MSU-induced inflammation.

In summary, we confirm that corilagin inhibits the NLRP3 inflammasome and pyroptosis by protecting mitochondrial integrity, reducing mitochondrial ROS production, and preventing the interaction between TXNIP and NLRP3. In addition, we have proved that corilagin can reduce inflammatory cytokines expression and immune cell infiltration during acute gouty arthritis flares (Figure 9). These results provide a reliable pharmacological basis for preclinical studies. As one of the active ingredients of natural medicine, the safety of corilagin is also guaranteed. Therefore, corilagin possesses excellent potential to treat NLRP3-dependent inflammatory diseases such as gout, diabetes, sepsis, and cancer.

## Figures and Tables

**Table 1:** The source and identifier of antibodies used in this study.

Antibodies	Source	Identifier
Anti-NLRP3	Cell signaling technology	Cat# 15101
Anti-ASC	Cell signaling technology	Cat# 67824
Anti-TXNIP	Cell signaling technology	Cat# 14715
Anti-Phospho (p)-I*κ*B*α*	Cell signaling technology	Cat# 2859
Anti-Phospho (p)-NF-*κ*B p65	Cell signaling technology	Cat# 3033
Anti-I*κ*B*α*	Cell signaling technology	Cat# 4814
Anti-NF-*κ*B p65	Cell signaling technology	Cat# 8242
Normal rabbit IgG	Cell signaling technology	Cat# 2729
Anti-rabbit IgG (Alexa Fluor^®^ 488 conjugate)	Cell signaling technology	Cat# 4412
Anti-mouse IgG (Alexa Fluor^®^ 555 conjugate)	Cell signaling technology	Cat# 4409
Anti-*β* actin antibody	Abcam	Cat# ab8227
Anti-GAPDH antibody	Abcam	Cat# ab181602
Anti-GSDMD antibody	Abcam	Cat# ab209845
Anti-NEK7 antibody	Abcam	Cat# ab133514
Anti-IL-1*β* antibody	Abcam	Cat# ab9722
Anti-*α*-tubulin antibody	Proteintech	Cat# 11224-1-AP
Anti-NLRP3 mouse mAb	Adipogen life sciences	Cat# AG-20B-0014-C100
Anti-caspase-1 (p20)	Adipogen life sciences	Cat# AG-20B 0042
Anti-caspase-1 antibody	Novus biologicals	Cat# NB100-56565
Ly-6G antibody	Thermo fisher scientific	Cat# 14-5931-82
F4/80 antibody	Thermo fisher scientific	Cat# 14-4801-82

## Data Availability

All data supporting the findings of this study were shown in this manuscript and its supplementary materials.

## Supplementary Materials

Supplementary Materials Figure S1: Corilagin does not inhibit TLR4/NF-*κ*B signaling pathway. Figure S2: NLRP3^−/−^ BMDMs do not secrete IL-1*β*. Figure S3: Corilagin does not affect the binding of NKE7 and NLRP3. Figure S4: Corilagin inhibits NLRP3 inflammasome activation independent of PKA or Nrf2 signaling.
