# Supplementary material for: Corilagin Restrains NLRP3 Inflammasome Activation and Pyroptosis through the ROS/TXNIP/NLRP3 Pathway to Prevent Inflammation
Source: Oxid Med Cell Longev. 2022 Oct 17;2022:1652244. doi: 10.1155/2022/1652244 (PMC9592212; doi:10.1155/2022/1652244)
Supplement: Supplementary Materials — Figure S1: Corilagin does not inhibit TLR4/NF-κB signaling pathway. Figure S2: NLRP3−/− BMDMs do not secrete IL-1β. Figure S3: Corilagin does not affect the binding of NKE7 and NLRP3. Figure S4: Corilagin inhibits NLRP3 inflammasome activation independent of PKA or Nrf2 signaling. [file 1652244.f1.docx]

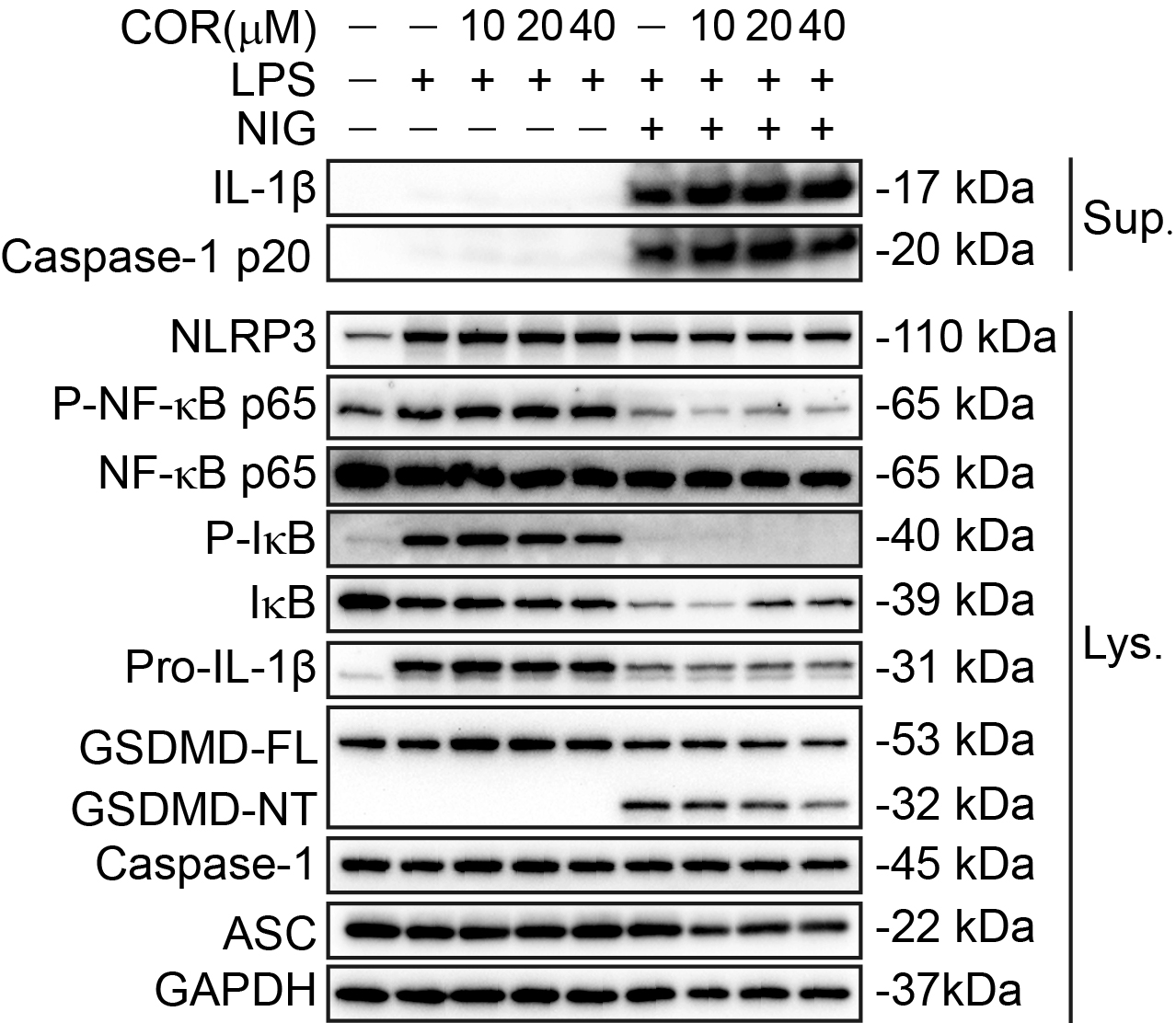


Figure S1: Corilagin does not inhibit TLR4/ NF-κB signaling pathway. BMDMs were pretreated with corilagin for 30 min and stimulated with LPS (0.5 µg/ml) for 4 h, followed by challenge with or without nigericin. Immunoblotting analysis of phospho (p)-NF-κB p65 and NF-κB p65, p-IκB and IκB, NLRP3, ASC, caspase-1 and p20，pro-IL-1β and IL-1β, GSDMD. GAPDH as loading control.


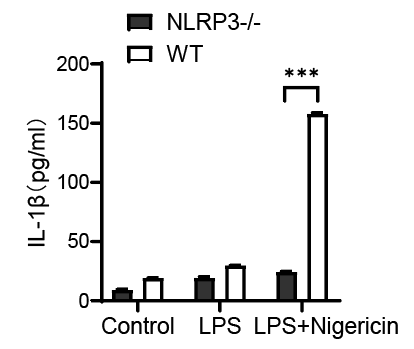


Figure S2: NLRP3-/- BMDMs do not secrete IL-1β. BMDMs from wild-type mice or NLRP3-/- mice were primed with LPS for 4 h and then stimulated with nigericin for 1 h. The IL-1β level in supernatant was detected by ELISA kit. ***P < 0.001.


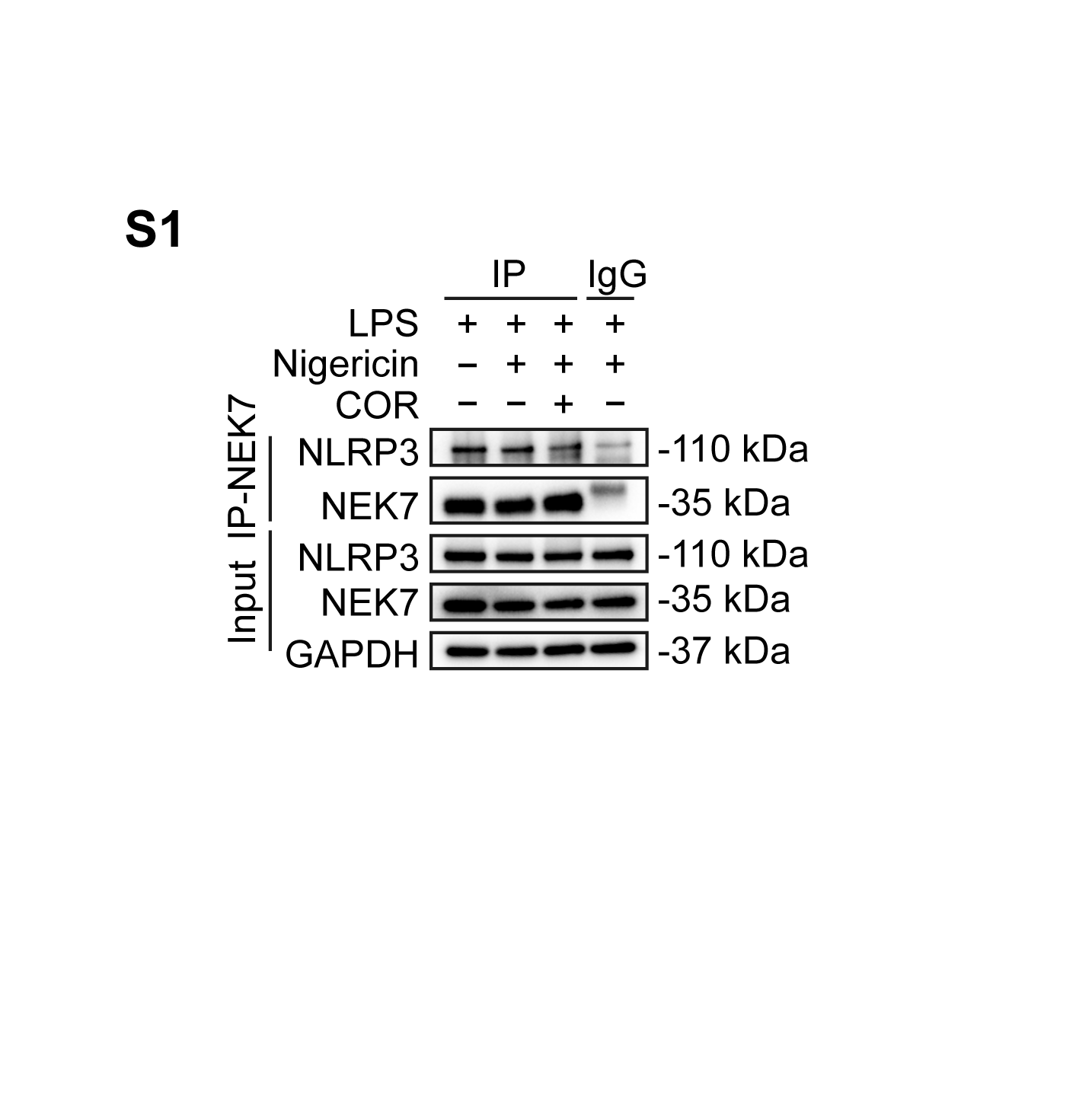


Figure S3: Corilagin does not affect the binding of NKE7 and NLRP3. BMDMs were primed with LPS for 4 h, followed by incubation with corilagin (40 μM) for 30 min, and then stimulated with nigericin for 1 h. The interaction between NEK7 and NLRP3 was analyzed by coimmunoprecipitation. COR, corilagin.


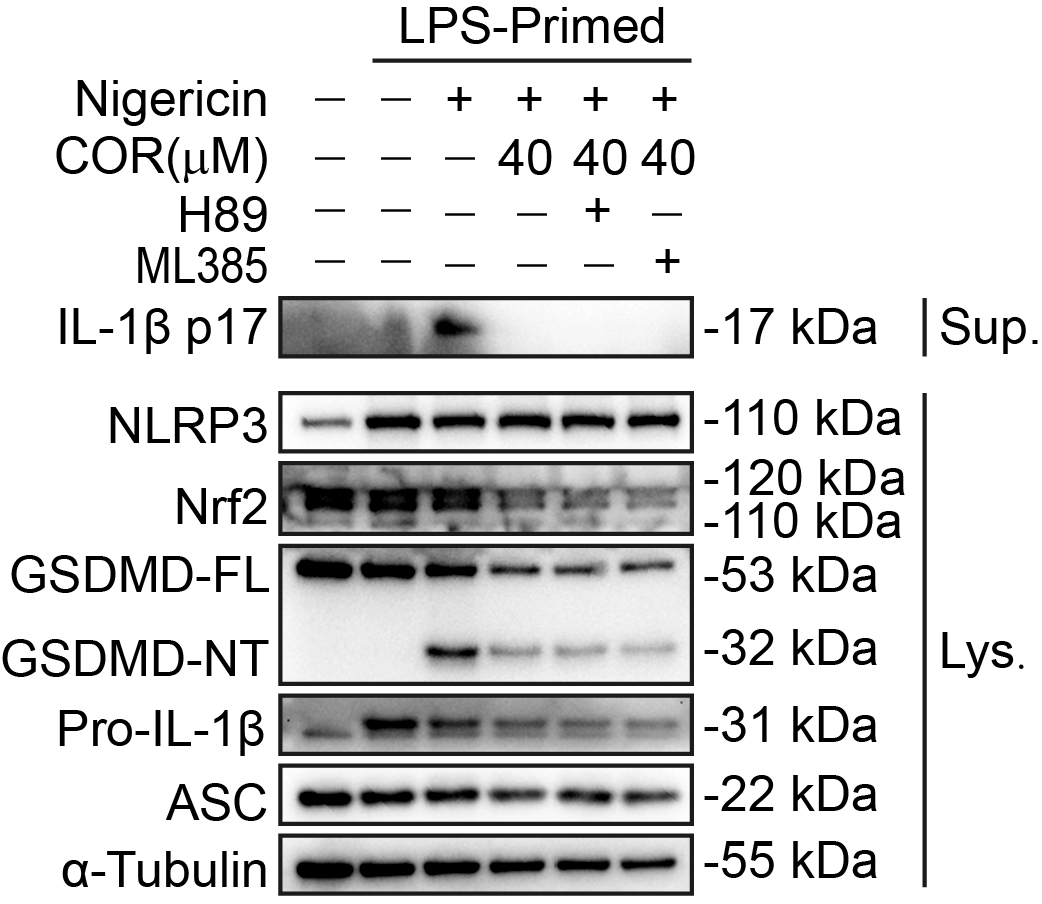


Figure S4: Corilagin inhibits NLRP3 inflammasome activation independent of PKA or Nrf2 signaling. LPS-primed BMDMs were incubated with a PKA inhibitor (10 μM H89) or Nrf2 inhibitor (2 μM ML385) in the presence of corilagin for 30 min followed by nigericin (1 h). Immunoblotting analysis of phospho (p)-NF-κB p65 and NF-κB p65, p-IκB and IκB, NLRP3, ASC, caspase-1 and p20，pro-IL-1β and IL-1β, GSDMD. α-Tubulin as loading control. COR, corilagin.
